# Supplementary material for: Deep learning exoplanets detection by combining real and synthetic data
Source: PLoS One. 2022 May 25;17(5):e0268199. doi: 10.1371/journal.pone.0268199 (PMC9132280; doi:10.1371/journal.pone.0268199)
Supplement: Attachment — Submitted filename: Review Response - PLoS.docx [file pone.0268199.s001.docx]

Response to Reviewer 1 Comments

**Point 1:** The authors have to list the research questions addressed by the proposed work and also the need for the proposed research.

**Response 1:**  Thank you for this comment. The main research questions addressed by this work are: How can artificial intelligence-based algorithms contribute to the exoplanet detection field, also if it is possible to add technical knowledge through synthetic data to improve the performance of the exoplanet detector.

The need of this research relies on three main aspects: First, the exoplanet detection as an opportunity to explore areas to look for other habitable worlds. Second, given many databases, the use of artificial intelligence to analyse massive data. Finally, the gaps that it aims to fill in the literature based on the previous works. Some examples of those gaps are the use of unconfirmed planets to train the models and the lack of evaluation of the models on unknown real data. To clarify this point, we modified the introduction.

**Point 2:** Xception architecture is used. But the authors did not explain the rationale behind choosing this model among many CNN models.

**Response 2:** Thank you for this comment. To clarify this point, we modified the detection model subsection.

**Point 3:** The need for and applications of the proposed work to be discussed.

**Response 3:** Thank you for this comment. The applications and future work extend the study to systems with more than one confirmed planet or planetary candidate dealing with multi-transit detection on the same light curve. Also, the implementation of the method on a different database like NASA’s Transiting Exoplanet Survey Satellite (TESS), the mission has discovered already 450 166 exoplanets and has 4604 planet candidates or even the data acquired from the James Webb Space Telescope launched in December 2021. To clarify this point, we modified the introduction and conclusion.

Response to Reviewer 2 Comments

**Point 1:** "The best ratio of synthetic data is determined by the performance of an optimization technique and a sensitivity analysis." Check grammar.

**Response 1:** Thank you for this comment. To clarify this point, we modified the abstract. We also performed a complete check of the paper and asked for a grammar, format, and style revision from experts.

**Point 2:** Highlight the significance of the proposed method with quantitative results.

**Response 2:** Thank you for this comment. The experimental results section performs three quantitative analyses: A heuristic approach to find a small range to narrow down the search for the amount of synthetic data that improves the detection model. Second, a fine adjustment to get the actual values of S and T. Finally, a metric comparison between our approach and the work presented in the literature. In order to clarify this point, we modified the experimental results section.

**Point 3:** "Section presents some exoplanet detection 67 approaches that can be found in the literature and briefly describe the approach which 68 is the start point of this work. Section details the proposed method. The section shows the 69 experimental results and a comparison with previous results. Finally, Section 70 summarizes the main conclusions and future work." Section numbers are missing.

**Response 3:** Thank you for this comment. To clarify this point, we modified the paper overview in the introduction.

**Point 4:** Justification for synthetic data generation need to be elaborately given.

**Response 4:** Thank you for this comment. The main purpose of synthetic data generation is to take advantage of the technical knowledge acquired from experts of the periodicity and modelling of the planet's transit and translate it into synthetic data that can be added to the model. To clarify this point, we modified the synthetic data generation subsection.

**Point 5:** Table 4 "Encode S" description is missing.

**Response 5:** Thank you for this comment. To clarify this point, we modified the Table 4 content.

**Point 6:** The pseudocode of GA related to the given problem must be included with an explanation.

**Response 6:** Thank you for this comment. We add the pseudocode of GA to the experimental results section**.**

**Point 7:** Only GA is used for comparison. How do other meta-heuristic algorithms like PSO, ACO, BA etc., perform?

**Response 7:** The main purpose of the heuristic search on this approach was to find a small range to narrow down the search for the values of S and T to obtain the highest possible F1 value. Those values are used to perform a fine adjustment described in the fourth section. We select GA based on its great performance and easy implementation. Given the nature of the optimization problem, we expect that other meta-heuristic algorithms find the same range of S and T. To clarify this issue, we modified the GA description.

**Point 8:** Description of the CNN model used is required.

**Response 8:** Thank you for this comment. To clarify this point, we modified the Detection model subsection.

**Point 9:** Citation to figures 1 and 2 is missing.

**Response 9:** Thank you for this comment. We already checked and both figures are cited in the text.
